# Impact of renal denervation on renalase expression in adult rats with spontaneous hypertension

**DOI:** 10.3892/etm.2012.616

**Published:** 2012-06-20

**Authors:** WEIHONG JIANG, YUNZHONG GUO, LIHUA TAN, XIAOHONG TANG, QIONG YANG, KAN YANG

**Affiliations:** Department of Cardiology, Third Xiangya Hospital, Central South University, Changsha, Hunan 410013, P.R. China

**Keywords:** hypertension, renal denervation, renalase, tyrosine hydroxylase

## Abstract

The aim of this study was to investigate the impact of renal denervation on the blood pressure, plasma renalase content and expression of renalase and tyrosine hydroxylase (TH) in the kidney of spontaneous hypertensive (SH) rats and to explore the mechanism of renal denervation involved in lowering blood pressure. SH rats (n=48) were randomly assigned to baseline, surgery (renal denervation), sham and control groups. WKY rats matched in age (n=12) served as the baseline control group. All rats were housed until they were 12 weeks old. The rats in the baseline group and the WKY group rats were sacrificed, and blood and kidney were collected for examination. In the renal denervation, sham and control groups, the blood pressure was continuously monitored. One and six weeks after renal denervation, 6 rats in each group were sacrificed, and blood and kidney were collected for examination. ELISA was employed to measure the plasma renalase, and western blot analysis was performed to detect the expression of TH and renalase in the kidney. Compared with the WKY rats, SH rats in the baseline group had significantly increased blood pressure and markedly elevated TH protein expression (P<0.05), but dramatically reduced plasma renalase content and protein expression of renalase in the kidney (P<0.05). One week after surgery, the mean arterial pressure and TH protein expression in the surgery group was lowered compared with the baseline group and dramatically reduced when compared with the sham and control groups (P<0.05). In the surgery group, renalase levels were markedly increased compared with the baseline, sham and control groups (P<0.05). Six weeks after renal denervation, the mean arterial pressure and TH levels in the surgery group were significantly increased while the renalase content and expression were markedly reduced compared with those at week 1, however, there were no marked differences among the surgery, sham and control groups (P>0.05). Moreover, no pronounced differences in the above variables were found between the sham and control groups at any timepoint (P>0.05). Renal denervation can lower blood pressure, which may be attributed to the suppression of sympathetic nerves, increase in plasma renalase content and renalase expression in the kidney.

## Introduction

Hypertension is caused by multiple factors. The kidney is an organ that plays an important role in the regulation of blood pressure. The hyperactive sympathetic nervous system in the kidney has been found to be an important cause of hypertension. Since the early 1900s, researchers have attempted to explore the non-drug treatment of hypertension via blocking the sympathetic nervous system in the kidney. In a multicenter, randomized control study in 2010 (1), researchers applied radiofrequency ablation of sympathetic nerves aiming to treat refractory hypertension. The results demonstrated the safety and effectiveness of interventional therapy of hypertension. However, to date, the blood pressure-lowering effect of renal denervation has not been confirmed and whether another mechanism apart from suppression of the sympathetic nervous system is involved is yet unclear. In the present study, the blood pressure, plasma renalase content and expression of renalase and tyrosine hydroxylase (TH) in the kidney were detected before and after renal denervation aiming to explore the mechanism of renal denervation in lowering blood pressure.

## Materials and methods

### Animals and main reagents

Spontaneously hypertensive rats (n=48) and age-matched WKY rats (n=12) weighing 240–280 g were purchased from Beijing Vitalriver Experimental Animal Co., Ltd. Animals were housed until they were aged 12 weeks and then used for experiments. The ELISA kit for renalase detection (Wier Biotech Co., Ltd), renalase antibody (Abcam), TH antibody (Minipo, USA) and goat anti-rabbit IgG (Proteintech, USA) were used in the present study.

### Grouping

i) The baseline control group, 12 WKY rats aged 12 weeks, were sacrificed before which the blood pressure was measured. Then, the blood and kidney were collected for examination. ii) The baseline group, male SH rats aged 12 weeks (n=12), were sacrificed before which the blood pressure was measured. Then, the blood and kidney were collected for examination. iii) The surgery (renal denervation) group, male SH rats aged 12 weeks (n=12), received renal denervation. iv) The sham group, male SH rats aged 12 weeks (n=12), underwent laparotomy but not renal denervation. v) The control group, male SH rats aged 12 weeks (n=12), were not treated.

In the surgery, sham and control groups the blood pressure was measured. One and six weeks following surgery, 6 animals in each group were sacrificed at each timepoint and the blood and kidney were collected for examination.

### Surgical procedure

Rats were anesthetized intraperitoneally at room temperature with chloral hydrate at 25 mg/kg. After sterilization, a midline incision was made in the abdomen followed by exposure of the subcutaneous tissues and the kidneys. The ureters and the arteries, veins and nerves in the sheath were observed. After stripping the arterial/venous sheath, the renal nerve was exposed followed by denervation under a microscope (magnification, ×25). Then, 10% phenol in 95% ethanol was used to treat the tissues around the veins. In the sham group, laparotomy was performed and the sympathetic nerve was exposed and treated with normal saline. The wound was subsequently closed, and rats were allowed to wake spontaneously. Within 3 days following surgery, intraperitoneal injection of 16 U of penicillin was administered once daily for prevention of infection.

### Measurement of blood pressure

A non-invasive blood pressure measuring instrument was used to measure the blood pressure at the tail artery. At room temperature, the rested rats were fixed on a table, and the balloon of the measuring instrument was inserted into the proximal end of the tail artery and the pulse transducer in the abdomen of the rats. The temperature was then increased to 39°C to completely dilate the tail artery. The other end of the instrument was connected to a computer. When the collected signals became stable, the balloon was inflated until the pulse signal became a straight line followed by deflation. When the pulse signals formed regular waves, the blood pressure was calculated with the software. Blood pressure was measured every 3 min which was performed three times and the mean was calculated.

### Detection of plasma renalase content in the kidney by ELISA

Washing solution was added to each well followed by vortexing for 30 sec, and then the washing solution was removed. These procedures were performed 5 times. Chromogenic reagent A (50 μl) and B (50 μl) were consecutively added to each well followed by vortexing and incubation in the dark at 37°C for 15 min. Then, stop solution (50 μl) was added to each well followed by incubation for 15 min (blue to yellow). The optical density (OD) was measured at 450 nm, and the standard curve was delineated. The renalase content was calculated according to the standard curve.

### Detection of renalase and TH expression by western blot analysis

The kidney was collected from SH rats in the baseline group and the WKY rats in the baseline control group and SH rats in the sham, surgery and control groups 1 and 6 weeks after surgery. The kidney was homogenated in RIPA followed by extraction of total protein. The protein concentration was detected with the Bradford method, and proteins were stored at −80°C. The proteins underwent denaturation for 5 min and were then subjected to SDS-PAGE for 3 h. Then, the proteins were transferred onto a PVDF membrane (90 min for TH; 60 min for renalase) which was blocked in 5% non-fat milk in TBST at room temperature for 1 h. The membrane was incubated with an anti-TH (1:500) or anti-renalase antibody (1:800) at 4°C overnight and then with a secondary antibody (1:3,000) at room temperature for 1 (TH) or 2 h (renalase). Following visualization, Quatity One software was employed to determine the optical density of the bands.

### Statistical analysis

SPSS version 17.0 was employed for statistical analysis. Quantitative data were expressed as means ± standard deviation (SD). Normal distribution was tested before comparisons. Comparisons of means between two groups were carried out with Student's t-test and those among different groups with one-way analysis of variance. A P-value <0.05 was considered to indicate a statistically significant result.

## Results

### General data

There were no significant differences in the heart rate and body weight among the surgery, sham and control groups before and after surgery (P>0.0).

### Blood pressure and plasma renalase of the SH rats in the baseline group and WKY rats

In the SH rats, the systolic blood pressure (SBP), diastolic blood pressure (DBP) and mean arterial pressure (MAP) were markedly higher than those in the WKY rats (SBP, 198±29 vs. 140±11 mmHg; DBP, 144±24 vs. 80±9 mmHg; MAP, 163±23 vs. 100±9 mmHg, P<0.05 for all). SH rats had a markedly reduced plasma renalase content when compared with the WKY rats (113.8±10.4 vs. 133.0±6.7 μg/ml, P<0.05) (Table I).

### Effect of renal denervation on MAP and plasma renalase content

Prior to surgery, there were no pronounced differences in the MAP and plasma renalase content among the surgery, sham and control groups (P>0.05). One week after the surgery, the MAP in the surgery group was markedly reduced (131±12, 164±9 and 163±7 mmHg, respectively; P<0.05) while the plasma renalase content was dramatically increased (127±5.1, 111.7±3.4 and 112.5±5.8 ng/l, respectively; P<0.05) when compared with the sham and control groups. Six weeks after surgery, no pronounced differences were found in the MAP and plasma renalase content among the three groups (P>0.05). In addition, the MAP and plasma renalase content in the sham group were comparable to those in the control group before and after surgery (P>0.05) (Table II).

### Expression of TH and renalase in the kidney in the different groups

**Figure 1:**
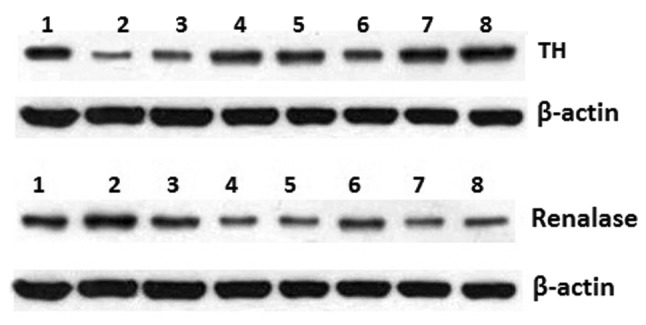
TH and renalase protein expression in the kidney of rats in the different groups. Lane 1, SH rats in baseline group; lane 2, WKY rats; lane 3, surgery group at week 1; lane 4, sham group at week 1; lane 5, control group at week 1; lane 6, surgery group at week 6; lane 7, sham group at week 6; lane 8, control group at week 6.

When compared with the WKY rats, the TH expression in the kidney of SH rats of the baseline group was markedly increased while the renalase expression dramatically reduced (P<0.05). One week after surgery, the TH expression in the surgery group was markedly reduced and lower than that in the sham and control groups (P<0.05), while the renalase expression was dramatically increased (P<0.05). Six weeks after surgery, the TH expression increased while the renalase expression was reduced as compared to these values at week 1 but both were comparable to those in the sham and control groups (P>0.05). There was no significant difference in the expression of TH and renalase between the sham or control groups and the baseline group. In addition, no marked difference was noted between the sham and control groups and between before and after surgery in both groups (P>0.05) (Fig. 1 and Table III).

## Discussion

The activity of the renal sympathetic nervous system is a determinant to the occurrence and development of hypertension. TH is a rate-limiting enzyme in the synthesis of catecholamine and can reflect the activity of focal sympathetic nerves. Burgi *et al* (4) found that TH expression in the kidney of SH rats was higher than that in age-matched WKY rats and was positively related to blood pressure. Our results showed that TH expression in the kidney and blood pressure of SH rats in the baseline group were higher than those of the WKY rats, which was consistent with the findings of Burgi *et al* (4). This demonstrated that TH expression can reflect the relationship between the sympathetic nervous system and blood pressure.

The renalase protein consists of a secretory signal peptide, a flavin adenine dinucleotide-binding region, and an amine oxidase domain. Renalase can degrade catecholamine, regulate blood pressure and protect the myocardium and has been an important factor in the field of heart and kidney research. Basic research demonstrated that reduction in renalase contributes to the occurrence of hypertension (3,4). Studies revealed that renalase is related to primary hypertension. In renalase-deficient mice, the SBP, DBP and catecholamine in the plasma and urine were found to be markedly increased (5–7). After injection of renalase siRNA, the renalase expression was markedly reduced accompanied by a significant increase in blood pressure, which suggests that renalase deficiency contributes to an increase in blood pressure (8). In the present study, when compared with the age-matched WKY rats with normal blood pressure, the renalase expression in the plasma and kidney was markedly reduced in the SH rats, which was consistent with previously reports and further confirmed the close relationship between renalase deficiency and hypertension.

In recent years, renalase has been found to be closely related to the activity of the renal sympathetic nerves and blood pressure (9). Clinical and animal studies (8,10,11) have shown that the increased activity of the sympathetic nerves and elevation of blood pressure are usually accompanied by reduction in renalase expression, and that renalase deficiency may cause an increase in blood pressure and activity of the sympathetic nerves. In the present study, the SH rats receiving renal denervation were used for experiments one week after surgery. Results showed that MAP and TH expression in the kidney were markedly reduced in the SH rats one week after surgery while these values in the sham and control groups were continuously maintained at a high level, which suggested that renal denervation resulted in reduction in the activity of renal sympathetic nerves and that the surgery was successful. In addition, our findings also revealed that the plasma renalase content and renalase expression in the kidney one week after surgery were dramatically higher than those in the sham and control groups. This indicates that renal denervation lowers blood pressure via affecting renalase expression.

The nerves in the kidney have important physiological functions. To date, the mechanism of renal denervation in lowering blood pressure is still unclear and renalase may be a participant in this mechanism. Xu *et al* (3) intravenously administered renalase to SD rats, and the results showed a reduction in SBP, DBP and MAP in these rats. This was also confirmed by our findings. However, the long-term effect of renal denervation on hypertension is still poorly understood. As shown in our findings, 6 weeks after surgery, blood pressure and TH expression increased to a certain extent in SH rats, while the plasma renalase content and renalase expression in the kidney reduced to a nearly normal level. Although differences were found between the surgery and sham groups or the control group, statistical significance was absent due to the small sample size. The cause of this finding is still unclear. Following renal denervation, the autocrine, paracrine or systemic sympathetic nervous system participates in this feedback, or the nerve regeneration affects the therapeutic efficacy. Thus, in future studies, a larger sample size is required to confirm the findings of the present study.

Taken together, in SH rats, the increased activity of the renal sympathetic nerves and deficient secretion of renalase are involved in the pathogenesis of hypertension, and the blocking of renal sympathetic nerves by denervation may increase the renalase expression in the kidney and the blood pressure. Our findings demonstrate that renalase is involved in the reduction of blood pressure following renal denervation. In recent years, studies have focused on the development of recombinant or synthetic renalase as a novel blood pressure-lowering drug (12), which may provide a new strategy for the treatment of hypertension and other cardiovascular diseases.

## Figures and Tables

**Table I:** Blood pressure and plasma renalase of SH rats in the baseline group and WKY rats (mean ± SD).

Group (n=12)	SBP (mmHg)	DBP (mmHg)	MAP (mmHg)	Renalase (μg/ml)
SHR	198±29^a^	144±24^a^	163±23^a^	113.8±10.4^a^
WKY	140±11	80±9	100±9	133.0±6.7

a P<0.05 vs. the WKY group (baseline control group). DBP, diastolic blood pressure; MAP, mean arterial pressure.

**Table II:** MAP and plasma renalase content before and after renal denervation (mean ± SD).

	MAP (mmHg)			Renalase (μg/ml)		
Group	Before	1 week	6 weeks	Before	1 week	6 weeks
Surgery	165±7	131±12^a–c^	167±4	113.8±5.4^a,b^	127±5.1^a–c^	114.8±6.6
Sham	165±8	164±9	170±6	111.9±3.7	111.7±3.4	112.1±7.8
Control	162±8	163±7	168±5	112.8±6.0	112.5±5.8	113.4±3.8

a P<0.05 vs. sham;

b P<0.05 vs. control;

c P<0.05 vs. before surgery.

**Table III:** Expression of TH and renalase in the kidney in the different groups (mean ± SD).

			Surgery group		Sham group		Control group	
Group	WKY rats	SH rats in baseline group	1 week	6 weeks	1 week	6 weeks	1 week	6 weeks
TH	0.241±0.02	0.492±0.04^a^	0.312±0.02^b–d^	0.478±0.01	0.507±0.03	0.538±0.01	0.499±0.04	0.555±0.02
Renalase	0.698±0.01	0.403±0.05^a^	0.608±0.01^b–d^	0.455±0.01	0.401±0.03	0.405±0.01	0.408±0.02	0.406±0.01

a P<0.05 vs. WKY rats;

b P<0.05 vs. SH rats in baseline group;

c P<0.05 vs. the sham group;

d P<0.05 vs. the control group.
